# Reviewer acknowledgement 2012

**DOI:** 10.1186/1742-4690-10-17

**Published:** 2013-02-27

**Authors:** Kuan-Teh Jeang

**Affiliations:** 1 The National Institutes of Health, Bethesda, MD, USA

## Abstract

**Contributing reviewers:**

*Retrovirology* would like to thank the following for their assistance with peer review of manuscripts for the journal in 2012.

## 

Marlen Aasa-Chapman

United Kingdom

Isabella Abbate

Italy

Christopher Aiken

United States of America

Lorraine Albritton

United States of America

Jose Alcami

Spain

Neil Almond

United Kingdom

Zandrea Ambrose

United States of America

Marie-Line Andreola

France

Nathalie Arhel

France

Eric Arts

United States of America

Eugene Asahchop

Canada

Masanori Baba

Japan

Eran Bacharach

Israel

Jan Balzarini

Belgium

Charles Bangham

United Kingdom

Norbert Bannert

Germany

Eric Barklis

United States of America

Stéphane Basmaciogullari

France

Jean-Luc Battini

France

Ali Bazarbachi

Lebanon

Anne-Sophie Beignon

France

Serge Benichou

France

Monsef Benkirane

France

Yamina Bennasser

France

Edward Berger

United States of America

Ben Berkhout

Netherlands

Clarisse Berlioz-Torrent

France

Neil Berry

United Kingdom

Lionel Berthoux

Canada

Edouard Betsem

France

Joel Blankson

United States of America

Ronald Bontrop

Netherlands

Kathleen Boris-Lawrie

United States of America

Persephone Borrow

United Kingdom

Fadila Bouamr

United States of America

Charles Boucher

Netherlands

Katarzyna Bozek

Germany

Ineke Braakman

Netherlands

Simon Brackenridge

United Kingdom

Abraham Brass

United States of America

Jason Brenchley

United States of America

Bluma Brenner

Canada

Laurence Briant

France

Mark Brockman

Canada

Christopher Broder

United States of America

James Brooks

Canada

Doryen Bubeck

United Kingdom

Shilpa Buch

United States of America

Michael Bukrinsky

United States of America

Donald Burke

United States of America

Dennis Burton

United States of America

Edward Campbell

United States of America

Mary Campbell

United States of America

Paula Cannon

United States of America

Mary Carrington

United States of America

Carol Carter

United States of America

Chris Case

United States of America

Hannah Castro

United Kingdom

Francesca Ceccherini-Silberstein

Italy

Shengxi Chen

United States of America

Irvin Chen

United States of America

Benjamin Chen

United States of America

Cecilia Cheng-Mayer

United States of America

Peter Cherepanov

United Kingdom

Andrea Cimarelli

France

Paul Clapham

United States of America

Janice Clements

United States of America

Kathleen Clouse

United States of America

Alan Cochrane

Canada

John Coffin

United States of America

Eric Cohen

Canada

Pierre Corbeau

France

Marion Cornelissen

Netherlands

Rebecca Craven

United States of America

Gael Cristofari

France

Atze Das

Netherlands

Siddhartha Datta

United States of America

Miles Davenport

Australia

Andrew Dayton

United States of America

Anthony De Vico

United States of America

Michele Di Mascio

United States of America

Felipe Diaz-Griffero

United States of America

Dimiter Dimitrov

United States of America

Robert Doms

United States of America

Margherita Doria

Italy

Alexei Drummond

New Zealand

Michel Dupon

France

Michael Emerman

United States of America

Stephane Emiliani

France

Alan Engelman

United States of America

Oliver Fackler

Germany

Ariberto Fassati

United Kingdom

Benoit Favier

France

Mark Federspiel

United States of America

Jacques Fellay

Switzerland

Dean Follmann

United States of America

Genoveffa Franchini

United States of America

Pierre Frange

France

Christophe Fraser

United Kingdom

Eric Freed

United States of America

Sebastian Fugmann

United States of America

Masahiro Fujii

Japan

Jun-ichi Fujisawa

Japan

Dana Gabuzda

United States of America

Vitaly Ganusov

United States of America

J. Victor Garcia

United States of America

Henrik Garoff

Sweden

Jeremy Garson

United Kingdom

Hiroyuki Gatanaga

Japan

Anne Gatignol

Canada

Glen Gaulton

United States of America

William Gelbart

United States of America

Antoine Gessain

France

Mauro Giacca

Italy

Nicolas Gilbert

France

Paul Goepfert

United States of America

Stephen Goff

United States of America

Nilu Goonetilleke

United Kingdom

Robert J. Gorelick

United States of America

Paul Gorry

Australia

Matthias Gotte

Canada

Heinrich Gottlinger

United States of America

Xavier Graña

United States of America

Luuk Gras

Netherlands

Patrick Green

United States of America

Jay Grobler

United States of America

John Guatelli

United States of America

Lutz Guertler

Germany

Samuel Gunderson

United States of America

Beatrice Hahn

United States of America

Nancy Haigwood

United States of America

William Hall

Ireland

David Harrich

Australia

P Richard Harrigan

Canada

Reuben Harris

United States of America

Hideki Hasegawa

Japan

Jonathan Heeney

United Kingdom

Thierry Heidmann

France

Christopher Hellen

United States of America

Joris Hemelaar

United Kingdom

Joshua Herbeck

United States of America

Georges Herbein

France

Vanessa Hirsch

United States of America

Philip Hogg

Australia

Thomas Hope

United States of America

Laurent Houzet

United States of America

James Hoxie

United States of America

Peter Hraber

United States of America

Wei-Shau Hu

United States of America

Stephen Hughes

United States of America

Peter Hunt

United States of America

Eric Hunter

United States of America

Hendrik Huthoff

United Kingdom

Tatsuhiko Igarashi

Japan

Sergey Iordanskiy

United States of America

Yukihito Ishizaka

Japan

Jacques Izopet

France

Richard Jenner

United Kingdom

Hezhao Ji

Canada

Shibo Jiang

China

Dong-Yan Jin

Hong Kong

Marc Johnson

United States of America

Paul Jolicoeur

Canada

Clare Jolly

United Kingdom

Rolf Kaiser

Germany

Ganjam Kalpana

United States of America

Aditi Kanhere

United Kingdom

Jonathan Karn

United States of America

Fatah Kashanchi

United States of America

David Katz

United Kingdom

Brandon Keele

United States of America

Paul Kellam

United Kingdom

Stephen Kent

Australia

Oliver Keppler

Germany

Rosemary Kiernan

France

Baek Kim

United States of America

Jason Kimata

United States of America

Frank Kirchhoff

Germany

Nichole Klatt

United States of America

Renate Koenig

Germany

Wayne Koff

United States of America

Donald Kohn

United States of America

Neeltje Kootstra

Netherlands

Yoshio Koyanagi

Japan

Christine Kozak

United States of America

Bjorn Kuhl

United States of America

Peter Kwong

United States of America

Bernard Lafont

United States of America

Nadine Laguette

France

Jean-Marc Lanchy

United States of America

Nathaniel Landau

United States of America

Michael Laughrea

Canada

Ha Youn Lee

United States of America

Andrew Lever

United Kingdom

Laura Levy

United States of America

Sharon Lewin

Australia

Yuxing Li

United States of America

Chen Liang

Canada

Yi Liu

United States of America

J. Stephen Lodmell

United States of America

Jeremy Luban

United States of America

Renaud Mahieux

France

Johnson Mak

Australia

Frank Maldarelli

United States of America

Michael Malim

United Kingdom

Fabrizio Mammano

France

Nicolas Manel

France

Anne-Genevieve Marcelin

France

Alessandro Marcello

Italy

Richard Markham

United States of America

David Markovitz

United States of America

Mark Marsh

United Kingdom

Julio Martin-Garcia

United States of America

Juan Martin-Serrano

United Kingdom

Bernard Masquelier

France

Tetsuro Matano

Japan

Masao Matsuoka

Japan

Wendy Maury

United States of America

Ernest Maynard

United States of America

Myra McClure

United Kingdom

Aine McKnight

United Kingdom

Mark McNally

United States of America

Gregory Melikyan

United States of America

Jean-Michel Mesnard

France

Karin J. Metzner

Switzerland

Masaaki Miyazawa

Japan

Takayuki Miyazawa

Japan

Trine Mogensen

Denmark

Ronald Montelaro

United States of America

John Moore

United States of America

Lynn Morris

South Africa

Donald Mosier

United States of America

Marylene Mougel

France

Andrew J Mouland

Canada

Viktor Müller

Hungary

Matteo Negroni

France

Stuart Neil

United Kingdom

Sergei Nekhai

United States of America

Yoshiaki Nishimura

United States of America

Sebastien Nisole

France

Marc Noguera-Julian

Spain

Philip Norris

United States of America

Vlad Novitsky

United States of America

David OConnor

United States of America

Paula Ordonez Suarez

United Kingdom

Mary Pacold

United States of America

Luis A Pardo

Germany

Roger Paredes

Spain

Vinay Pathak

United States of America

William Paxton

Netherlands

Martine Peeters

France

Jean-Marie Peloponese

France

Yuri Persidsky

United States of America

Boris Matija Peterlin

United States of America

Claudine Pique

France

Mauro Pistello

Italy

Massimo Pizzato

Italy

Eric Poeschla

United States of America

Stefan Pöhlmann

Germany

Georgios Pollakis

Netherlands

Yves Pommier

United States of America

Art Poon

Canada

Vinayaka Prasad

United States of America

Flor Pujol

Venezuela

Lynn Pulliam

United States of America

Miguel Quinones-Mateu

United States of America

Alan Rein

United States of America

Axel Rethwilm

Germany

Andrew Rice

United States of America

Yolande Richard

France

Marjorie Robert-Guroff

United States of America

Olivier Rohr

France

Monica Roth

United States of America

Jean-Pierre Routy

Canada

Sarah Rowland-Jones

United Kingdom

Jonah Sacha

United States of America

Ali Saib

France

Mineki Saito

Japan

Jun-ichi Sakuragi

Japan

Marco Salemi

United States of America

Rogier Sanders

Netherlands

Stefanos Sarafianos

United States of America

Quentin Sattentau

United Kingdom

Bassel E Sawaya

United States of America

Celia Schiffer

United States of America

Michael Schindler

Germany

Olivier Schwartz

France

Walter Scott

United States of America

Jamie Scott

Canada

Oliver John Semmes

United States of America

Xiaoying Shen

United States of America

Marc Sitbon

France

Jacek Skowronski

United States of America

Nicolas Sluis-Cremer

United States of America

Thomas Smithgall

United States of America

Ellen Sparger

United States of America

Paul Spearman

United States of America

Dave Speijer

Netherlands

Klaus Strebel

United States of America

Hendrik Streeck

United States of America

Michael Summers

United States of America

William Switzer

United States of America

Chetan Tailor

Canada

Toshikazu Takeshita

Japan

Yasuhiro Takeuchi

United Kingdom

Masafumi Takiguchi

Japan

Amalio Telenti

Switzerland

Alice Telesnitsky

United States of America

Markus Thali

United States of America

Kenzo Tokunaga

Japan

Georgia Tomaras

United States of America

Greg Towers

United Kingdom

Simon Travers

South Africa

Klaus Überla

Germany

Susana Valente

United States of America

Tonja van der Kuyl

Netherlands

Carine Van Lint

Belgium

Koen Van Rompay

United States of America

Anne-Mieke Vandamme

Belgium

Linos Vandekerckhove

Belgium

Sue vandeWoude

United States of America

Sundararajan Venkatesan

United States of America

Bruno Verhasselt

Belgium

Gabriel Wagner

United States of America

Abdul Waheed

United States of America

Mark Wainberg

Canada

Xiaohua Wang

United States of America

Robin Weiss

United Kingdom

Kirsten White

United States of America

James Whitney

United States of America

Brian Wigdahl

United States of America

Lucas Willems

Belgium

Kenneth Witwer

United States of America

Joseph Wong

United States of America

Li Wu

United States of America

Yuntao Wu

United States of America

Gutian Xiao

United States of America

Hongtao Xu

Canada

Masahiro Yamashita

United States of America

Xiaojian Yao

Canada

Junichiro Yasunaga

Japan

Ahmad Yatim

France

Man Lung Yeung

Hong Kong

John Young

United States of America

Jerome Zack

United States of America

Osvaldo Zagordi

Switzerland

Richard Y Zhao

United States of America

Yong-Hui Zheng

United States of America

Zhi-Ming Zheng

United States of America

Joseph Ziegelbauer

United States of America

Michael Zwick

United States of America

## Author’s information

Dr Kuan-Teh Jeang passed away on January 27^th^ 2013. This article was seen and approved by him in early January 2013.

